# Coronary plaque instability assessed by positron emission tomography and optical coherence tomography

**DOI:** 10.1007/s12149-021-01651-2

**Published:** 2021-07-17

**Authors:** L. Galiuto, L. Leccisotti, G. Locorotondo, I. Porto, F. Burzotta, C. Trani, G. Niccoli, A. M. Leone, M. L. Danza, V. Melita, E. Fedele, A. Stefanelli, A. Giordano, F. Crea

**Affiliations:** 1 Department of Cardiovascular and Thoracic Sciences, Fondazione Policlinico Universitario Agostino Gemelli IRCCS, Università Cattolica del Sacro Cuore, Largo A. Gemelli 8, 00168 Rome, Italy; 2 Nuclear Medicine Unit, Fondazione Policlinico Universitario Agostino Gemelli IRCCS, Rome, Italy; 3 Nuclear Medicine Institute, Università Cattolica del Sacro Cuore, Rome, Italy

**Keywords:** FDG, Innovative biotechnologies, OCT, PET, Plaque inflammation, Plaque instability

## Abstract

**Background:**

Non-ST-elevation myocardial infarction (NSTEMI) and unstable angina (UA) are caused often by destabilization of non-flow limiting inflamed coronary artery plaques. ^18^F-fluorodeoxyglucose (FDG) uptake with positron emission tomography/computed tomography (PET/CT) reveals plaque inflammation, while intracoronary optical coherence tomography (OCT) reliably identifies morphological features of coronary instability, such as plaque rupture or erosion. We aimed to prospectively compare these two innovative biotechnologies in the characterization of coronary artery inflammation, which has never been attempted before.

**Methods:**

OCT and FDG PET/CT were performed in 18 patients with single vessel coronary artery disease, treated by percutaneous coronary intervention (PCI) with stent implantation, divided into 2 groups: NSTEMI/UA (*n* = 10) and stable angina (*n* = 8) patients.

**Results:**

Plaque rupture/erosion recurred more frequently [100% vs 25%, *p* = 0.001] and FDG uptake was greater [TBR median 1.50 vs 0.87, *p* = 0.004] in NSTEMI/UA than stable angina patients. FDG uptake resulted greater in patients with than without plaque rupture/erosion [1.2 (0.86–1.96) vs 0.87 (0.66–1.07), *p* = 0.013]. Among NSTEMI/UA patients, no significant difference in FDG uptake was found between ruptured and eroded plaques. The highest FDG uptake values were found in ruptured plaques, belonging to patients with NSTEMI/UA. OCT and PET/CT agreed in 72% of patients [*p* = 0.018]: 100% of patients with plaque rupture/erosion and increased FDG uptake had NSTEMI/UA.

**Conclusion:**

For the first time, we demonstrated that the correspondence between increased FDG uptake with PET/CT and morphology of coronary plaque instability at OCT is high.

## Introduction

Acute coronary syndromes (ACS) represent one of the main causes of death worldwide. While in stable coronary artery disease (CAD) myocardial ischemia worsens with growing stenosis severity, in most cases of ACS rupture or erosion occur at the site of non-flow limiting coronary artery plaques. Increased inflammatory state determines coronary artery plaque instability leading to ACS [1]: indeed, inflammatory cell infiltrates have been found in culprit plaques by histology. Presence of activated macrophages, plaque fissure and endothelial denudation with superficial platelet aggregation are considered major criteria for plaque instability [2, 3]. The development of innovative imaging biotechnologies targeted at these features could lead to the in vivo identification of such high-risk plaques in vivo and guide the development of novel personalized treatment strategies. Optical Coherence Tomography (OCT) currently represents the most promising invasive imaging technique to characterize coronary artery plaques, thanks to its capability to discriminate between smooth fibrous cap, plaque rupture or erosion with or without macrophage infiltration [4]. By performing OCT, it has been recently demonstrated that patients with ACS, having plaque rupture as mechanism of coronary instability, have a worse prognosis when compared with those having an intact fibrous cap. Conversely, patients with intact fibrous cap exhibit different mechanisms of instability, including thrombus at the site of plaque erosion, or intense vasoconstriction of epicardial arteries or coronary microcirculation [5]. Positron emission tomography/computed tomography (PET/CT) with ^18^F-fluoro-deoxy-glucose (FDG) has been tested to assess atherosclerotic plaque instability. Plaque glycolysis detected by FDG PET can be used as a surrogate marker of plaque inflammation and hypoxia. FDG uptake has been found greater in aortic and left main coronary artery plaques of patients with ACS and specifically in lesions judged responsible for ACS compared with lesions associated with stable coronary syndromes [6].

No data currently exist describing direct comparison between OCT and FDG PET/CT about the characterization of coronary artery plaques. In the present study, we aimed to prospectively evaluate the correlation between FDG PET/CT and OCT imaging tools in the assessment of coronary artery plaques of patients with ACS compared to a control group with stable angina.

## Methods

### Study population

The present study was designed as a single-center prospective cohort study, to compare OCT and FDG PET/CT tools in the evaluation of coronary artery plaque inflammation. Patients consecutively admitted to our department from 2012 to 2014 with ACS (either non-ST elevation myocardial infarction, NSTEMI, or unstable angina, UA) or stable angina, were considered for enrolment. Patients with NSTEMI/UA had prolonged chest pain at rest and electrocardiographic ischemic changes with or without elevation of cardiac necrosis biomarkers [7]. Patients with stable angina (control group) manifested exertional chest pain together with instrumental evidence of inducible myocardial ischemia (i.e., onset of angina and/or ST-segment depression ≥ 1 mm during exercise stress test, or ST-segment depression ≥ 1 mm and evidence of inducible left ventricular regional wall motion abnormalities, with or without angina, during stress echocardiography). Above mentioned clinical presentations were taken into account as reference to allocate patients into the two study groups, since no OCT or PET/CT methods are currently considered gold standard imaging tools in the evaluation of coronary artery inflammation**.** According to guidelines, coronary angiography with OCT examination and percutaneous coronary intervention (PCI) was planned within 48 h from admission. In all patients, OCT was performed just before PCI with stent implantation. PET/CT was planned within 24 h after PCI. The following inclusion criteria had to be satisfied: (1) single vessel CAD suitable to PCI; (2) no previous myocardial revascularization; (3) no need of pre-dilatation before crossing target lesion with OCT image wire. Patients were excluded if they had: (1) very high risk profile at admission; (2) severe left ventricular contractile dysfunction (i.e., left ventricular ejection fraction ≤ 30%); (3) renal failure, insulin-dependent diabetes and pregnancy; (4) platelets concentration < 100.000/mm^3^; (5) any contraindication to antiaggregant/anticoagulant or contrast agents. Study protocol was approved by the local Ethics Committee and was carried out according to Declaration of Helsinki on human rights. Written informed consent was obtained from each patient.

### OCT assessment

A frequency-domain OCT was employed. M2 LightLab OCT wire, characterized by an outer diameter of 0.019″ and containing a 0.006″ fibre-optic imaging core (less than 0.4 mm in diameter) and a distal radiopaque spring tip, was used, thus providing images with a longitudinal resolution of 15 μm. A length of at least 25 mm, comprising target lesion and proximal and distal reference segments, was chosen for a meaningful assessment of the coronary plaque. Once the image wire was positioned in the target vessel, it was pulled back at 2 mm/sec speed, during simultaneous manual infusion of a viscous isomolar infusion (Iodixanol) at an infusion rate of 1–3 cc/sec. All OCT frames were analysed off-line, in a validated core laboratory by a single expert interventional cardiologist (G.N.), unaware of clinical data. Data about signs of plaque rupture and erosion, and presence of macrophages were collected. Moreover, presence of lipid core was determined and thickness of fibrous cap was measured for comparison between NSTEMI/UA and stable angina patients.

### FDG PET/CT imaging

To minimize physiological FDG myocardial uptake, all patients were asked to observe a very low carbohydrate, high protein and high fat diet the day before PET imaging and then to fast overnight on the day before imaging. Pre-scan glucose levels were lower than 120 mg/dl in all patients. PET/CT imaging was performed with a dedicated 3D PET/CT scanner (Gemini GXL 16, Philips) 2 h after the intravenous administration of 3 MBq/Kg body weight of FDG. This protocol represents the best compromise between ALARA principle, low blood pool background signal and an acceptable duration of PET imaging [8], although FDG dose is lower than that used in previous studies [6, 21]. Imaging protocol included a scout followed by non-contrast-enhanced CT scan of thorax (100 kV and 50 mA) for attenuation correction and anatomical localization. PET imaging of the heart was then performed in list-mode for 15 min with cardiac gating. Patients were instructed to breathe normally during both CT and PET. After dead time, scatter, random and decay correction, PET data were reconstructed using an iterative algorithm based on the row-action maximum-likelihood algorithm (3D-RAMLA) with and without attenuation correction.

### FDG PET/CT analysis

Summed PET and CT images were fused and analysed by two experienced readers (L.L. and A.S.), blinded for all information regarding patient characteristics and classification, on a dedicated workstation (Extended Brilliance Workspace, Philips Healthcare). First, the alignment of CT and PET was reviewed to check proper co-registration using sternum, aorta, myocardium and vertebral bodies as major landmarks. In all cases, CT and PET alignment was found to be optimal. Each scan was subsequently assessed for the degree of FDG myocardial suppression near the stent (0 = minimal FDG myocardial uptake, 1 = mild FDG myocardial uptake, 2 = moderate FDG myocardial uptake, 3 = intense FDG myocardial uptake; a score ≥ 2 was considered inadequate suppression) as well as for coronary FDG uptake measurement. Coronary plaque maximal standardized uptake value (SUVmax, the decay corrected tissue concentration of the tracer divided by the injected dose per body weight) was measured after checking the perfect PET and CT co-registration in each stented coronary vessel: nine anterior descending arteries (ADA), six right coronary arteries (RCA) and three left circumflex arteries (LCA). The stent site was identified on the CT images and used to guide placement of 5-mm^2^ regions of interest (ROIs) over the coronary segment. The SUVmax value of at least two ROIs was recorded and the mean value was calculated. Coronary activity was only quantified where myocardial uptake could be confidently avoided. Moreover, the blood pool activity was determined by placing a VOI sphere of 1cm^3^ in the center of right atrium. For each coronary segment, a target-to-background ratio (TBR) was calculated by dividing coronary SUVmax to venous blood pool SUVmean.

### Statistical analysis

SPSS software package for Windows 17.0 (SPSS Inc., Chicago Illinois) was used. The absence of any cut-off value for FDG uptake within inflamed plaques reported in the literature prevented us to perform an analysis of intended sample size. Nevertheless, number of patients initially screened for enrolment was greatly higher than that previously reported in the literature. Data were expressed as median with interquartile range (IQr) or as percentage, for continuous or categorical variables, respectively. Differences between NSTEMI/UA and stable angina groups were tested by Mann–Whitney *U* test or Chi square test, respectively for continuous and categorical variables. Overall difference in TBR values between groups was assessed by Kruskal–Wallis test. ROC curve analysis was performed to set a cut-off value of TBR, corresponding to NSTEMI/UA setting. Concordance between OCT and PET/CT data was expressed by Kappa for agreement. A *p* value < 0.05 was considered for statistical significance throughout the study. No undetermined neither missing data entered statistical analysis. Analyses of variability in diagnostic accuracy was not done in the present study because PET/CT methodology used in the present study was already validated [6]. Intra-class correlation coefficients (ICC) with 95% confidence interval (CI) were calculated for inter-observer variability using TBR values obtained by the readers during the analysis.

## Results

**Fig. 1:**
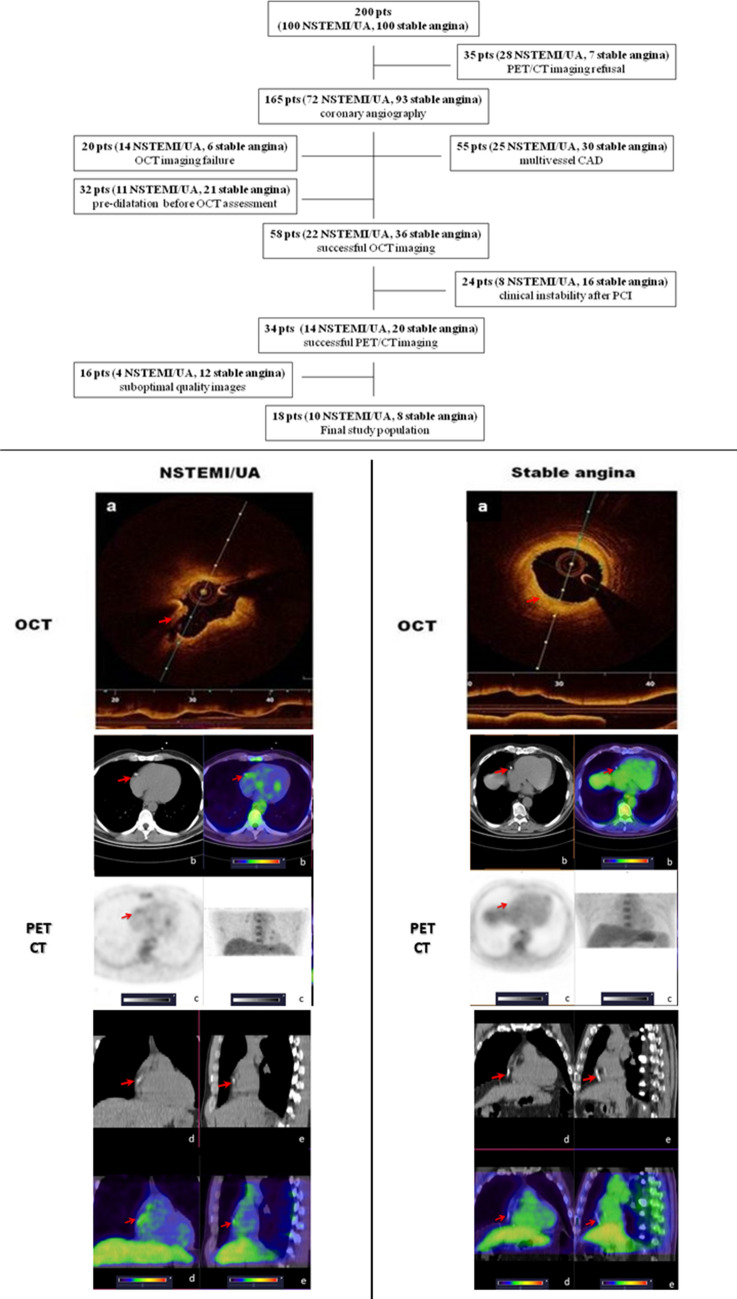
The *upper* part of the figure shows flow-chart of the study. In the *bottom*, representative images of OCT and FDG PET/CT techniques are displayed, for comparison between morphological appearance and functional feature of right coronary artery plaques in a patient with NSTEMI (left panel) and stable angina (right panel). *LEFT*: OCT (**a**) illustrate irregular surface of atherosclerotic plaque with zone of rupture corresponding to increased FDG uptake at the level of right coronary artery on axial (**c**), coronal (**d**) and sagittal (**e**) fused images; Target to Background ratio (TBR) = 1.42. *RIGHT*: OCT (**a**) illustrate regular atherosclerotic plaque with smooth surface. FDG PET/CT (**b**–**e**) images show not significant FDG uptake at the level of right coronary artery on axial (**c**), coronal (**d**) and sagittal (**e**) fused images; TBR = 0.90

**Fig. 2:**
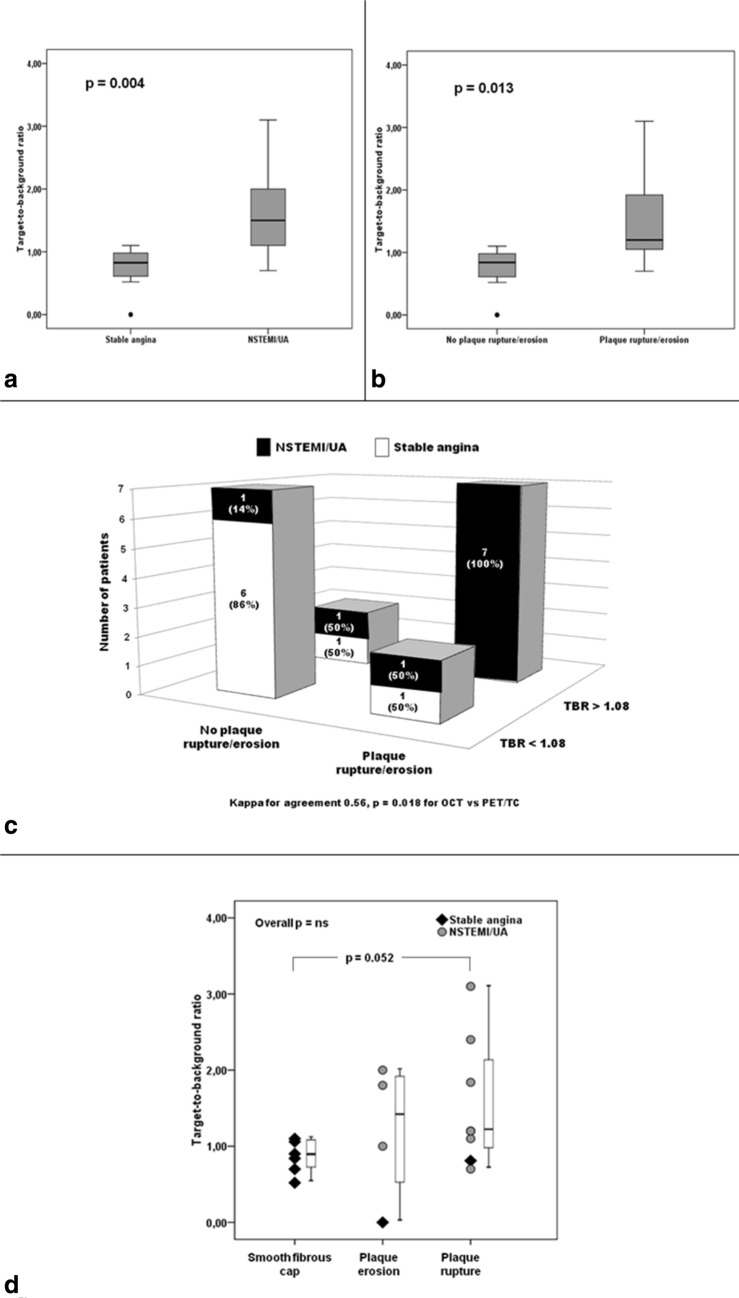
Differences in FDG uptake, expressed as Target-to-Background (TBR) values, between patients with stable angina and patients with NSTEMI/UA (panel **a**), and between plaques displaying rupture/erosion and plaques without rupture/erosion at OCT (panel **b**). In panel **c**, patients were distributed basing on TBR values and OCT data. By considering 1.08 cut-off value of TBR, capable to distinguish unstable clinical setting, a good agreement with OCT characteristics is found. Concordance between the two imaging tools correctly reflected clinical setting in more than 70% of cases. Panel **d** shows distribution of TBR values in the overall study population. Entire population is grouped basing on type of plaques (plaque with smooth, eroded or ruptured fibrous cap) and clinical setting: a trend towards highest values in ruptured plaques, most of them belonging to patients with NSTEMI/UA, is displayed

Out of 200 patients initially screened for enrolment, 35 patients (28 with NSTEMI/UA and 7 with stable angina) refused to undergo PET/CT scan. One hundred and seven patients were excluded for various reasons: 20 patients (14 with NSTEMI/UA and 6 with stable angina) because of OCT imaging failure, 55 patients (25 with NSTEMI/UA and 30 with stable angina) due to presence of multivessel coronary artery disease, and 32 patients (11 with NSTEMI/UA and 21 with stable angina) because they underwent balloon dilation of coronary artery before OCT assessment. Among the remaining 58 patients (22 with NSTEMI/UA and 36 with stable angina) successfully treated by PCI, 34 (20 with NSTEMI/UA and 14 with stable angina) could not perform PET/CT because of acute clinical instability, and 6 patients (5 with NSTEMI/UA and 1 with stable angina) were subsequently excluded by data analysis due to inadequate myocardial FDG suppression (score ≥ 2). Thus, only 18 patients (10 with NSTEMI/UA and 8 with stable angina) represented the final study population (Fig. 1, upper). OCT was performed at 1.6 ± 0.8 days and at 2 ± 0.5 days after admission, respectively in NSTEMI/UA and in stable CAD groups (*p* = 0.57). PET/CT was performed at 1.9 ± 0.4 days and 2.5 ± 0.7 days after OCT and PCI, respectively in NSTEMI/UA and in stable CAD groups (*p* = 0.11). Representative OCT and FDG PET/CT images are shown in Fig. 1, bottom. Characteristics of the overall study population are reported in Table 1, while imaging features for each enrolled patient are detailed in Table 2. No differences between groups were noted in cardiovascular risk profile, neither in prevalence of diseased coronary arteries. In all patients with disease of RCA, proximal-mid segments were involved. Most of patients were taking statin on admission, due to dyslipidaemia or carotid plaque. After admission, in patients not already under treatment, statin therapy was started, so that all enrolled patients were treated by statins throughout the study until discharge. A significant difference in white blood cell count on admission was found between NSTEMI/UA and stable CAD patients (*p* = 0.015). Plaque rupture was more frequent in patients with NSTEMI/UA than in those with stable angina (*p* = 0.015) (Table 1). No differences were detected about presence of erosion, lipids and thickness of fibrous cap between groups. Taking together, a higher frequency of plaque rupture/erosion was found in patients with NSTEMI/UA than in those with stable angina (*p* = 0.001). Conversely, out of eight patients with stable angina, six did not show any rupture/erosion, so that concordance of OCT imaging with clinical setting was significant (*p* = 0.001). Among the 18 analysed patients, physiological FDG uptake in the myocardium was minimal in 12 (67%) patients, mild in 4 patients (22%), moderate or intense in 2 (11%) patients. All stented coronary segments were evaluable by PET/CT because spillover of adjacent myocardium could be confidently avoided. In the two patients with inadequate myocardial suppression, FDG uptake was not near the stent. There was no significant difference in the degree of myocardial uptake among patient groups (*p* = ns) as well as in venous blood pool SUVmean (1.2 ± 0.2 vs 1.01 ± 0.2 in stable vs NSTEMI/UA, *p* = 0.17). Coronary FDG uptake of stented lesions, expressed as TBR, was significantly greater in patients with NSTEMI/UA than in those with stable angina (*p* = 0.004) [Fig. 2a]. A drug-eluting stent (DES) was implanted in all patients (8, 100%) in stable angina group and in seven (70%) patients in NSTEMI/UA group, while a bare-metal stent (BMS) was implanted in only three (30%) patients in NSTEMI/UA group (*p* = 0.091). Taking together, values of TBR did not significantly differ between plaques implanted with DES [1.1 (0.84–1.8)] and with BMS [1.8 (1–2)] (*p* = 0.426). Moreover, by performing analysis only in NSTEMI/UA group, we did not find any difference in TBR values between DES and BMS (*p* = 0.667). Notably, TBR values directly correlated with white blood cell count (Rho 0.56, *p* = 0.015), and inversely correlated with fibrous cap thickness (rho = − 0.56, *p* = 0.016), whereas no significant correlations were found with lipid arc (*p* = 0.86) and length (*p* = 0.18) (Table 3). Moreover, no difference in TBR values was noted, regarding to presence or absence of macrophages (*p* = 0.77).

**Table 1:** Clinical characteristics of study population

	Total (*n* = 18)	NSTEMI/UA (*n* = 10)	Stable CAD (*n* = 8)	*p*
Clinical and biochemical characteristics				
Age, years (median, IQr)	62 (44–87)	62 (47–83)	62 (59–65)	ns
Male sex, *n* (%)	16 (88)	8 (80)	8 (100)	ns
Hypertension, *n* (%)	10 (55%)	5 (50)	5 (67)	ns
Diabetes, *n* (%)	4 (22)	3 (26)	1 (12)	ns
Dyslipidemia, *n* (%)	11 (61)	7 (59)	4 (45)	ns
Current or past tobacco use, *n* (%)	11 (61)	6 (52)	5 (66)	ns
Family history of CAD, *n* (%)	4 (22)	2 (18)	2 (29)	ns
Diseased coronary artery, *n* (%)				
ADA	10 (55.5)	5 (50)	5 (62.5)	ns
LCA	1 (5.5)	0	1 (12.5)	ns
RCA	7 (39)	5 (50)	2 (25)	ns
Statin therapy on admission, *n* (%)	14 (78)	9 (90)	5 (63)	ns
Biochemical evaluation				
White blood cell count, *n* × 10^9 (median, IQr)	6.1 (5.1–7.9)	7.8 (6.9–9.8)	5 (4.5–5.9)	**0.015**
Creatinine on admission, mg/dL, (median, IQr)	0.81 (0.78–1.1)	0.81 (0.76–1.1)	0.94 (0.80–1.1)	ns
Creatinine at discharge, mg/dL, (median, IQr)	0.80 (0.80–1.1)	0.80 (0.80–1.1)	0.93 (0.80–1.1)	ns
Imaging characteristics				
TBR, median (IQr)	1.10 (0.89–1.81)	1.50 (1.08–2.10)	0.87 (0.73–1.09)	**0.004**
Rupture, *n* (%)	8 (44)	7 (70)	1 (12.5)	**0.015**
Erosion, *n* (%)	4 (22)	3 (30)	1 (12.5)	ns
Macrophages, *n* (%)	12 (67)	8 (80)	4 (50)	ns
Cap Thickness (µm), median (IQr)	80 (60–115)	60.00 (57.50–72.75)	105 (62.50–117.50)	ns
Lipidic Plaque, *n* (%)	11 (61)	8 (80)	3 (37.5)	ns
Maximum lipid arc (µm), median (IQr)	172.5 (73–220)	187.5 (91–210)	120 (0–308.7)	ns
Length of the plaque (µm), median (IQr)	16 (11.2–20.7)	15 (11.7–20.2)	16.5 (10–22.7)	ns

*ADA* anterior descending coronary artery, *CAD* coronary artery disease, *IQr* interquartile range, *LCA* left circumphlex artery, *NSTEMI/UA* non-ST elevation myocardial infarction/unstable angina, *RCA* right coronary artery

**Table 2:** Clinical, imaging and stent details of enrolled patients

Patient	Clinical setting	Coronary artery	TBR	Rupture/erosion	Cap Thickness (µm)	Macrophages	Lipid arch	Length of plaque	Stent type
N.1	Stable CAD	ADA	0.9	No	110	Absent	NA	NA	DES
N.2	NSTEMI	ADA	1.2	Yes	60	Present	180	13	DES
N.3	Stable CAD	ADA	1.1	No	110	Absent	110	25	DES
N.4	Stable CAD	RCA	0.7	Yes	60	Present	NA	NA	DES
N.5	UA	ADA	1.84	Yes	65	Present	200	22	DES
N.6	Stable CAD	ADA	0.52	No	130	Present	130	10	DES
N.7	NSTEMI	RCA	1.1	Yes	60	Present	165	12	DES
N.8	Stable CAD	ADA	0.81	Yes	60	Present	305	10	DES
N.9	NSTEMI	ADA	3.1	Yes	67	Present	205	20	DES
N.10	Stable CAD	RCA	1.06	No	70	Present	320	18	DES
N.11	NSTEMI	RCA	1.8	No	90	Absent	100	11	BMS
N.12	UA	RCA	1	No	160	Present	225	17	BMS
N.13	NSTEMI	RCA	2	Yes	60	Present	300	21	BMS
N.14	NSTEMI	ADA	2.4	Yes	60	Absent	195	20	DES
N.15	NSTEMI	LCA	1.2	Yes	50	Present	64	13	DES
N.16	Stable CAD	ADA	0.84	No	160	Absent	0	15	DES
N.17	NSTEMI	LCA	1.8	No	110	Present	0	8	DES
N.18	Stable CAD	LCA	1.1	No	110	Present	0	22	DES

*ADA* anterior descending artery, *BMS* bare metal stent, *CAD* coronary artery disease, *DES* drug eluting stent, *LCA* left circumphlex artery, *NSTEMI* Non-ST segment elevation myocardial infarction, *RCA* right coronary artery, *TBR* target-to-background ratio, *UA* unstable angina

**Table 3:** Correlations between OCT features of plaque and TBR

	TBR	
	Rho	*p*
Cap thickness (µm)	− 0.56	0.016
Lipid arch	0.05	0.86
Length of plaque	0.35	0.18

A TBR cut-off value of 1.08 could be identified to distinguish unstable and stable clinical setting with 83.3% sensitivity and 87.5% specificity (AUC 0.92, *p* = 0.002). In the overall study population, TBR values were significantly higher in plaques displaying rupture/erosion than in those without rupture/erosion [1.2 (0.86–1.96) vs 0.87 (0.66–1.07), *p* = 0.013] [Fig. 2b]. However, no significant difference was found in TBR values between ruptured and eroded plaques (*p* = ns). By combining OCT data of plaque instability with TBR values, good agreement was found between the two imaging tools (*p* = 0.018) [Fig. 2c]: indeed, all plaques with both morphological and functional signs of coronary artery instability corresponded to an unstable clinical setting. Despite the absence of significant difference between plaques with smooth fibrous cap, plaques with erosion and plaques with rupture, a trend in FDG uptake was found [Fig. 2d], with lowest TBR values in plaques with smooth fibrous cap, highest TBR values in plaques with rupture and intermediate TBR values in plaques with erosion. Ruptured plaques, most of them in patients with NSTEMI/UA, displayed increased TBR, as compared to plaques with smooth fibrous cap (*p* = 0.052), which entirely belonged to patients with stable angina. For FDG PET/CT imaging, the ICC between readers was 0.77 (95% CI 0.65–0.9).

## Discussion

The results of this prospective study demonstrate that: (1) FDG uptake is higher in ACS (NSTEMI/UA) than in stable CAD and corresponds to morphological features of plaque instability, identified by OCT; (2) agreement between the two imaging tools is excellent and concordance with clinical setting is relevant. To the best of our knowledge, a direct comparison of FDG PET/CT with OCT imaging has never been attempted before. By expanding previous results, our findings further prove in patients that enhanced coronary plaque FDG uptake corresponds to anatomical features of plaque instability.

In the last decades, invasive and non-invasive innovative biotechnologies have been developed to characterize coronary plaque instability. Mechanisms leading to ACS may complicate plaques with variable characteristics [9]. Patients with ACS and evidence of plaque rupture by OCT show widespread features of vulnerability of the entire coronary circulation [5]. In our analysis, plaque rupture was more prevalent than plaque erosion, as reported in previous studies [10]. Our finding of plaque rupture/erosion in a minority of patients (two out of eight) presenting with stable angina is not surprising, as it is known that subclinical rupture is not infrequent in patients presenting with stable CAD [11]: in both these patients, however, TBR values were low. Notably, coronary arteries showing OCT features of instability presented increased inflammation, as compared to coronary arteries characterized by plaques with smooth fibrous cap. Inflammation plays an important role in driving plaque instability: it is a key factor driving the progression of atherosclerosis, the formation of the necrotic core and ultimately fibrous cap rupture. Since inflammatory cells consume large amounts of glucose compared with other plaque cells, PET imaging with FDG may display activity in atherosclerosis, potentially useful for identifying unstable plaques. It is important to recognize unstable plaques in clinically relevant beds (carotid and coronary arteries) to both refine risk prediction and guide novel treatment strategies targeted for primary prevention of ACS. The correlation of FDG uptake and atherosclerotic inflammation has been largely demonstrated in animal and human studies [12, 13], mainly in carotid artery plaque [14–17]. The increased FDG uptake of metabolically active plaques might depend on abundance of macrophages pro-atherogenic (M1) resident in the inflamed plaques [18, 19]. Macrophage proliferation has been documented in culprit lesions after plaque rupture, leading to myocardial infarction or sudden cardiac death [20]. PET imaging of coronary plaques has been reported and is significantly more challenging than that of carotids [13, 21, 22] because of the small size of coronary plaque, and the blurring effect caused by cardiac and respiratory motion. In an intriguing pilot study, Rogers et al. found greater FDG uptake in culprit lesions judged to be responsible for ACS (*n* = 10) compared with lesions associated with stable coronary syndromes (*n* = 15) thus demonstrating for the first time the feasibility of PET/CT in detecting coronary artery plaque inflammation [6]. However, some uncertainty about reliability of FDG PET/CT has been raised. In a study of Joshi et al. [17], FDG uptake was increased in the culprit vessel of only 33% of patients, with no significant differences between the culprit plaques and the remaining coronary vasculature, while ^18^F-sodium fluoride uptake was increased at the site of culprit ruptured plaques in 93% of ACS patients, with TBR values that were a third higher than the maximum activity anywhere else in the coronary tree. In that study, however, myocardial suppression of FDG uptake was achieved in 70% of ACS patients and coronary FDG uptake could not be distinguished from patchy myocardial uptake in 52% of vessel territories. The main differences of our results compared to already published data are the different imaging delay after revascularization, patient preparation, PET/CT imaging technique and the lack of coronary plaque characterization by OCT in previous studies. We performed PET/CT imaging within 24 h from PCI and according to the proposed protocol for FDG PET/CT imaging in atherosclerosis, optimized to keep as low as possible the blood signal [8].

It is well known that the accurate estimation of FDG uptake in coronary plaque is extremely challenging. In particular, FDG uptake measurement depends on the size of the plaque (volume of ~ 0.1 mL), the contrast due to the activity of inflammatory cells and the resolution of the scanner (~ 4 mm). Atherosclerotic lesions falling below the spatial resolution of the scanner are particularly at risk of partial volume effect. We did not use any method to improve the quantification of tracer activity, such as voxel-based adjustment after co-registration with CT with contrast. Firstly, we saved the patient an additional load of iodinated contrast medium and ionizing radiation. Secondly, partial volume correction by CT could be difficult to implement because the inflammatory part of the lesion cannot be seen by CT. Thirdly, we preferred to act on PET acquisition duration. This may modify the measured values to a small lesion: increasing the acquisition duration decreases the variability of the error for a given measure, especially for SUVmax. We therefore performed a 15-min bed and reconstruction including an iterative approach. Moreover, it should not be forgotten that the advantage of PET compared to other imaging modalities is its superior sensitivity, which allows the detection of picomolar tracer concentrations. Finally, for a given lesion, even if the measurement of FDG uptake is not correct, it still conveys useful information on functional features of coronary plaques. Despite the strong underestimation of FDG signal in coronary plaques, the measured SUVmax describes a combination of both activity concentration and lesion size. In our study, there is no reason to believe that the size of coronary lesions is significantly different in ACS compared to stable patients. Therefore, the significantly different FDG uptake reflects the different concentration of inflammatory cells within the plaques. FDG measurement within the coronary plaque may be limited by the “spill-in” from the blood pool within the ROI. However, this occurs in all plaques, regardless of type.

The localization of coronary FDG uptake is also difficult because of the small size of coronary artery plaque and coronary motion. Therefore, we used as reference point for coronary plaques the stent placed at the site deemed to be the lesion responsible for ACS or stable angina at the time of PCI. Ideally, FDG PET/CT imaging should be performed prior to PCI but it is not feasible in a real clinical setting as well as not ethical in ACS patients. In our study, all patients in both groups underwent the same invasive protocol, comprising left and right coronary angiography, OCT and subsequent stent implantation. Standardization of invasive protocol was required in the attempt to minimize any potential differences in FDG uptake induced by PCI-related trauma. Despite such similarities, FDG uptake signal was significantly different between the two groups. Thus, since every coronary plaque in NSTEMI/UA and stable angina groups were subjected to the same PCI-related trauma, it is conceivable that most of the difference between FDG signal observed in NSTEMI/UA vs stabile angina groups is related to plaque inflammation more than trauma. On the other hand, it could be hypothesized that our results reflect intrinsic properties of coronary plaques, not only related to inflammation itself, but also related to the individual inflammatory response of plaques to PCI. ECG-gated PET acquisition, performed according to imaging protocol, was not used for imaging analysis, as the loss of PET data signal was more significant than the advantage of reduced motion. However, improvements of respiratory and cardiac gating methods might be useful for coronary PET imaging [23, 24]. Regarding ECG gating for coronary PET imaging, instead of using counts obtained exclusively from the end-diastolic phase, all counts of the full cardiac cycle could be used, potentially avoiding the noise-amplification problem [25]*.* Our dietary recommendations resulted in adequate suppression of myocardial FDG uptake in 18/24 (75%) of patients. Our rate is comparable with that of previous studies (57%-85%) [6, 17, 21, 22] and could be further improved by intravenous pre-administration of unfractionated heparin to increase plasma free fatty acid levels [26]. Contrary to previous studies that enrolled patients with multivessel CAD, in the present study, we intentionally chose to assess only patients with single vessel CAD, to have certainty about correspondence between anatomical and functional imaging of coronary artery plaques at the culprit site. Indeed, it is known that FDG uptake is increased not only at the coronary culprit site, but also within the ascending aorta and coronary non-culprit sites [6, 17]. The absence of significant difference in TBR values between plaques with rupture and erosion is not surprising, as inflammatory cell activation is a key component in both cases, although macrophages play a key role in plaque fissure while neutrophils play a key role in erosion [9, 27, 28].

The principal limitation of our study is the small sample size, mainly secondary to the complexity of enrolment and imaging methods, which required good adherence of patients to protocol and rigorous timing and methodology. The small sample size could have affected our results. However, by performing a post hoc analysis of statistical power, according to the mean value of TBR in the two study groups, we found a power of 97.3%, and then a β error of 2.7%. This TBR cut-off value cannot be used to assess the instability of coronary plaques not undergoing PCI or in clinical practice. This is a pilot explorative study. These promising findings need to be validated in a larger prospective multicenter study, which also includes healthy subjects and aims to link coronary plaque FDG uptake to patient outcome. The lack of contrast CT angiography could be a further limitation. This would allow a greater co-registration of PET and CT. We did not perform CT angiography to limit patient radiation exposure. However, ECG and respiratory gating may clearer co-registration of PET data to the coronary arteries. Some clinical studies on the imaging of coronary plaque have been performed with ^18^F-FDG and/or ^18^F-NaF [17]. We used only FDG for the easiness of synthesis, the ready availability in our Centre and its routine use for several clinical applications. ^18^F-NaF is a promising tracer for coronary plaque imaging. Recently, Majeed et al. demonstrated that ^18^F-NaF uptake was associated with multiple high-risk plaque features detected by OCT in ACS patients, especially with plaque macrophage and cholesterol crystals [29]. However, to date it is not yet clear which of the possible radiotracers targeting different biological processes such as inflammation, micro-calcification, angiogenesis, matrix remodelling, apoptosis or hypoxia has the greatest chance for risk stratification of major cardiac events. Finally, although it is known that C-Reactive Protein (CRP) is increased in ACS and predict prognosis, we did not routinely tested it in clinical practice in all patients. Thus, we could not assess correlation between CRP levels and TBR values. However, our purpose was to test correspondence between different indicators of inflammation, rather than verify prediction of prognosis. In this regard, the significant correlation with white blood cell count confirms a strict relationship between imaging and biochemical expression of inflammation.

## Conclusions

The current study shows, for the first time in patients, that the anatomical features of plaque instability (rupture/erosion) assessed by OCT are associated with enhanced FDG uptake after PCI as detected non-invasively by PET/CT. Actually, TBR values did not differ between plaques with rupture and plaques with erosion, thus PET signal cannot discriminate between these two different presentations of coronary instability, and may be therefore inadequate to guide personalized forms of treatment. Surely these results are preliminary and need to be confirmed in larger trials.
